# Severe non-atopic prurigo nodularis in an otherwise healthy elderly patient: rapid multidimensional response to dupilumab

**DOI:** 10.3389/fphar.2026.1876166

**Published:** 2026-07-21

**Authors:** Alessandra Ventre, Federica Nuccio, Sebastiano Gangemi, Luisa Ricciardi

**Affiliations:** Allergy and Clinical Immunology Unit of G. Martino Hospital, Department of Clinical and Experimental Medicine, University of Messina, Messina, Italy

**Keywords:** biologic therapy, chronic pruritus, dupilumab, interleukin-13, interleukin-4, neuroimmune interaction, prurigo nodularis, type 2 inflammation

## Abstract

**Background:**

Prurigo nodularis (PN) is a chronic neuroimmune skin disorder characterized by intensely pruritic nodules, severe impairment of quality of life and significant psychosocial burden. Prior to the approval of targeted therapies, management relied on conventional agents with limited efficacy. Recent evidence of its neuroimmune pathogenesis have led to the approvement of targeted biologics. Dupilumab, a human monoclonal antibody targeting IL-4/IL-13-axis, has been the first targeted therapy approved for PN.

**Case presentation:**

We report a 75-year-old woman presenting with severe, treatment-naïve generalized pruritus (IGA PN-S 4; NRS 9/10; DLQI 21) with extensive nodular involvement of the trunk and extremities, complete sleep deprivation and important anxiety and depression (HADS.A 12; HADS-D 11). Following diagnosis of PN, treatment with dupilumab (600 mg loading dose, then 300 mg every 2 weeks) was initiated in October 2025. At approximately 6 weeks, marked pruritus reduction (NRS 3) and sleep restoration were observed. Progressive improvement continued over time. At 4 months follow-up, complete remission was nearly achieved (NRS 1, IGA PN-S 1, DLQI 2, HADS-A 4, HADS-D 3), with no adverse events reported.

**Conclusion:**

This case corroborates and extends the existing evidence on dupilumab efficacy in the treatment of moderate-to-severe PN, with particularly rapid and comprehensive responses across pruritus, skin lesions, sleep and psychosocial outcomes in an elderly patient, through IL-4/IL-13 inhibition. Our findings support dupilumab as a safe and effective targeted option for severe PN, also in older patients where conventional systemic therapies, such as corticosteroids or immunosuppressants, carry a high risk of adverse effects.

## 1 Introduction

Prurigo nodularis (PN) is a chronic skin disorder characterized by the presence of multiple, nodular lesions referred as intensely pruriginous. Subsequent scratching can further amplify the pruritus, resulting in a self-reinforcing itch-scratch cycle that leads to hyperkeratotic, excoriated or crusted lesions. These nodules are often symmetrically distributed, with a preferential localization at the extensor surfaces of the extremities and at the trunk. The severity can range from a few to several hundred in number and from 10 mm to 2–3 cm in size. Additional sensory phenomena such as stinging, burning, tingling, heat and/or cold are possible (Kwatra, 2022).

This condition is associated with a marked impairment in quality of life due to the severe and persistent itch, affecting patients’ sleep, work and social life, and significant psychosocial burden (Huang et al., 2020).

The real prevalence of PN remains difficult to establish since it is often underrecognized; it has been reported that it affects approximately 72 per 100.000 people, with a preferential involvement of middle-aged and older adults and darker phototypes (Kwatra, 2022). Association with atopic comorbidities, as well as systemic conditions including chronic renal disease, hepatic diseases, hematological malignancies and HIV infection has been described (Huang et al., 2020).

The pathogenesis of PN is complex and not yet fully elucidated, but it is increasingly recognized as a neuroimmune disorder resulting from the interplay between immune dysregulation and neuronal sensitization (Criado et al., 2024). The disease is primarily characterized by a type 2 inflammatory response with increased expression of interleukin (IL)-4, IL-13 and IL-31 in lesional skin. In addition, other inflammatory pathways, including IL-17 and IL-22, have been involved contributing in keratinocyte proliferation and barrier dysfunction (Liao et al., 2024). IL-4 and IL-13 promote Th2 polarization and enhance neuronal sensitivity to pruritogenic stimuli, while IL-31 directly activate sensory neurons, thus perpetuating the itch-scratch cycle (Shao et al., 2023). Neuronal dysregulation is another key feature of the disease, with evidence of altered cutaneous innervation and increased neural excitability. The neuroimmune crosstalk involves neuropeptides, such as substance P and calcitonin gene-related peptide (CGRP), released from nerve fibers that cause vasodilation and further recruitment of eosinophils and mast cells (Liao et al., 2024).

The histopathology of PN lesions is characterized by epidermal hyperplasia and marked hyperkeratosis and acanthosis. In the dermis, lesions are rich in infiltrates composed of lymphocytes, eosinophils, mast cells, along with overexpressed IL-4, IL-13 and IL-31. Reduction in nerve fiber density is typically observed (Liao et al., 2024).

In recent years, advances in the understanding of PN immunopathogenesis have paved the way for targeted biologic therapies. Among these, dupilumab has emerged as a key therapeutic strategy, thanks to its dual inhibiting action on IL-4/IL-13 axis (Yosipovitch et al., 2023).

Despite the growing evidence supporting dupilumab in prurigo nodularis, elderly patients remain relatively underrepresented in both clinical trials and real-world studies. This is clinically relevant because older patients may be particularly vulnerable to the adverse effects of conventional systemic therapies and often present with a substantial disease burden. Therefore, additional evidence regarding the effectiveness and safety of dupilumab in this population is warranted.

The aim of this report is to describe the clinical course of a 75-year-old woman with severe non-atopic prurigo nodularis treated with dupilumab and to highlight its impact on pruritus, skin lesions, sleep quality, quality of life, and psychological wellbeing during a 4-month follow-up period.

Herein, we report a case of PN successfully treated with dupilumab, with significant clinical improvement observed over a 4-month follow-up period.

## 2 Case description

A 75-year-old female patient was referred to our Allergy and Clinical Immunology Unit from the Emergency Department on 10 September 2025, for evaluation of a severe and diffuse pruritic skin eruption, which had started 2 months before, had initially been suspected to be of allergic origin, and had not improved under antihistamine oral treatment, with cetirizine 10 mg, and systemic corticosteroids, both betamethasone i. m. 4 mg injections and oral Prednisone 25 mg daily.

At presentation, the patient reported intense and persistent pruritus, described as continuous and particularly severe during nighttime. The severity of itching led to almost complete sleep deprivation, significantly impairing daily functioning and overall quality of life.

Baseline pruritus intensity, assessed using the Numerical Rating Scale (NRS), was 9/10. The Dermatology Life Quality Index (DLQI) score was 21, indicating an extremely large impact on quality of life. Psychological assessment revealed clinically relevant anxiety and depressive symptoms, likely secondary to chronic pruritus and sleep disturbance, with a Hospital Anxiety and Depression Scale (HADS) score of 12 for anxiety (HADS-A) and 11 for depression (HADS-D).

The patient had no relevant medical comorbidities and no personal history of atopic diseases.

Skin examination revealed widespread cutaneous involvement characterized by numerous hyperkeratotic, firm papules and nodules diffusely distributed over the trunk, with predominant involvement of the dorsal region. Lesions were frequently associated with excoriations, hemorrhagic crusts, and signs of chronic scratching. Several nodules appeared lichenified, and areas of post-inflammatory hyperpigmentation were observed. The distribution was symmetrical, mainly involving the upper and lower back, with higher lesion density in the lower dorsal region (Figure 1). The Investigator Global Assessment for Prurigo Nodularis–Stage (IGA PN-S) score was 4, consistent with severe disease.

**FIGURE 1:**
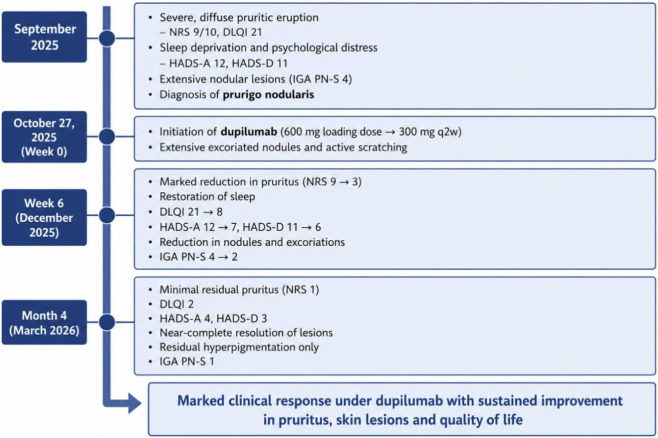
Timeline with relevant data from the episode of care, according to CARE case report guidelines.

Given the severity of the condition, the extent of cutaneous involvement, and the marked impairment in quality of life, treatment with dupilumab was initiated on 27 October 2025, with a 600 mg loading dose administered subcutaneously, followed by 300 mg every 2 weeks. The clinical course is summarized in Figure 1.

The patient was monitored through regular clinical follow-up and photographic illustration documenting a progressive transition from severe nodular disease to near-complete resolution (Figure 2). At treatment initiation, clinical images showed extensive disease activity with numerous excoriated nodules and papules diffusely distributed over the trunk, including the dorsal region and abdomen, as well as the upper and lower limbs, associated with active scratching (Figures 2A1–A4).

**FIGURE 2:**
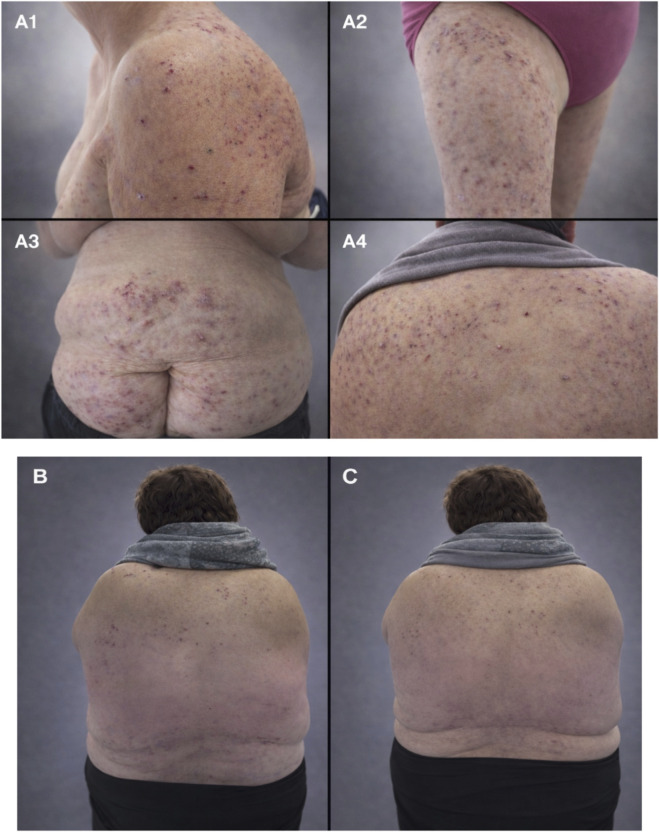
Clinical evolution of prurigo nodularis under dupilumab treatment. **(A)** Baseline: diffuse involvement with numerous excoriated nodules and papules affecting the trunk (including dorsal region and abdomen) and upper and lower limbs. **(B)** Week 6: marked reduction in inflammatory lesions and excoriations, with residual scattered papules. **(C)** Month 4: near-complete resolution of active lesions with residual post-inflammatory hyperpigmentation and minimal skin thickening.

At approximately 6 weeks (December 2025), a marked improvement in pruritus was observed, with NRS decreasing from 9/10 to 3/10. This was associated with restoration of sleep. DLQI improved from 21 to 8, and psychological assessment showed reduction in anxiety and depressive symptoms (HADS-A 12→7; HADS-D 11→6). Skin examination revealed a reduction in the number and size of nodules, decreased erythema, and fewer excoriations, with IGA PN-S improving from 4 to 2 (Figure 2B).

At approximately 4 months (March 2026), the patient reported minimal residual pruritus (NRS 1/10), no longer interfering with sleep or daily activities. DLQI further improved to 2, and HADS-A and HADS-D decreased to 4 and 3, respectively. Dermatological examination showed near-complete resolution of active lesions, with only residual post-inflammatory hyperpigmentation and mild skin thickening. The IGA PN-S score improved to 1, indicating almost clear skin (Figure 2C).

## 3 Diagnostic assessment

Routine laboratory investigations, including hematological and biochemical parameters, were within normal limits.

In addition to the diffuse nodular eruption, a localized lesion with well-defined margins was observed on the back, approximately palm-sized, previously diagnosed as dermatophytosis due to Trichophyton species. Although clinically distinct, this finding contributed to the initial diagnostic complexity.

The diagnosis of PN was established based on clinical findings, including chronic severe pruritus, the presence of multiple hyperkeratotic nodules with excoriations, and a characteristic distribution pattern. Alternative diagnoses, including allergic dermatoses and infectious causes, were considered and excluded based on clinical evaluation, laboratory findings and non-response to H1-antagonists and corticosteroids. Particular attention was paid to the differential diagnosis of other chronic pruritic dermatoses presenting with nodular lesions. In particular, nodular bullous pemphigoid was considered because of the patient’s age and the presence of severe pruritus. However, the patient had no history of blistering lesions, urticarial plaques, or other clinical features suggestive of bullous pemphigoid. Clinical examination consistently revealed multiple hyperkeratotic and excoriated nodules typical of prurigo nodularis, without evidence of vesicles or bullae at presentation or during follow-up. Furthermore, the overall clinical morphology and distribution of lesions were considered more consistent with prurigo nodularis. Therefore, bullous pemphigoid was considered unlikely on clinical grounds.

## 4 Discussion

This case report describes a 75-year-old patient with severe PN achieving near-complete disease control after 4 months of dupilumab.

Prior to the approval of targeted therapies, management of PN relied on conventional approaches including high-potency topical corticosteroids, topical calcineurin inhibitors, systemic antihistamines, systemic corticosteroids, immunosuppressants, neuromodulators and others. However, these agents often provide limited efficacy and high risk of adverse reactions or disease relapses (Biazus Soares and Yosipovitch, 2024).

Dupilumab is a fully human monoclonal antibody directed against the IL-4 receptor alpha subunit shared by the IL-4 and IL-13 receptor complexes inhibiting both inflammatory pathways. Dupilumab has demonstrated good efficacy and safety in other T2-driven inflammatory chronic diseases like atopic dermatitis (Beck et al., 2014), asthma (Castro et al., 2018), chronic rhinosinusitis with nasal polyps (Bachert et al., 2019), eosinophilic esophagitis (Dellon et al., 2022). The efficacy of Dupilumab in reducing itch intensity (as measured by the Worst Itch Numeric Rating Scale) and significantly improving typical skin lesions in PN affected patients, compared to placebo, has been demonstrated in pivotal phase III randomized controlled trials (LIBERTY-PN PRIME and PRIME2) (Yosipovitch et al., 2023).

Further subsequent real-world studies and systemic reviews have confirmed its efficacy and long-term safety (Cao et al., 2023; Zhao Y. et al., 2025), both in atopic and non-atopic populations (Kim et al., 2026). These findings have led to FDA approval in 2022 of dupilumab as the first targeted systemic therapy for PN (Vieira et al., 2025). More recently, another biologic such as Nemolizumab, a humanized monoclonal antibody targeting the IL-13 receptor A (IL-31RA), has been taken into consideration, since IL-31 is a neurocytokine directly involved in itch signaling and neuroimmune crosstalk (Vieira et al., 2025). Other therapeutic options for PN are at different stages of their development, including oral Janus kinase (JAK) 1 inhibitors and topical JAK1/2 inhibitors (Bianco et al., 2025).

The rapid and substantial reduction in itch intensity observed in our PN patient–from NRS 9/10 at baseline to 3/10 at week 6 and 1/10 at 4 months–is consistent with the findings of the pivotal phase 3 trials. In the LIBERTY-PN PRIME and PRIME2 trials a reduction of >/ = 4 point in WI-NRS at week 24 was achieved by 60% of dupilumab-treated patients in PRIME and by 37.2% in PRIME2, compared to 18.4% and 22% of placebo-treated patients, respectively (Yosipovitch et al., 2023). The pooled analysis of these two trials further confirmed these results and revealed earlier effect onset in itch: at week 24, 58.8% of dupilumab-treated patients achieved clinically meaningful improvement in itch, and 46.4% achieved clear or almost-clear skin, compared to 19.0% and 17.1% in the placebo group (p < 0.0001 for all) (Yosipovitch et al., 2024). Notably, our patient exceeded these average response rates, achieving near-complete pruritus resolution and IGA PN-S score of 1 (almost clear) at 4 months—a result that compares favorably even with the 52-week real-world cohort data reported by Zhao et al., where NRS scores decreased from a baseline of 7.65 ± 2.27 to 1.01 ± 0.65 and IGA scores from 3.53 ± 0.54 to 0.54 ± 0.33 by week 52 (Zhao Y. et al., 2025).

Our patient experienced a marked improvement in DLQI from 21 to 2 over 4 months, representing a notable result consistent with other real-world data: in a Swedish case series, significant improvements in DLQI scores were documented after 3 months of dupilumab treatment, from a median of 15 to 6 (p = 0.002) (Lönndahl et al., 2022). Similarly, the multicenter Chinese real-world study by Zhao et al. reported substantial improvement in patient-reported quality of life outcomes, with DLQI decreasing from 16.6 ± 6.8 at baseline to 6.4 ± 4.5 at week 16 (Zhao Z. et al., 2025). In our patient the degree of DLQI improvement at 4 months possibly reflects the durable suppression of the itch-scratch cycle.

In line with the finding of the pooled PRIME/PRIME2 analysis, in our case we registered a pronounced improvement in anxiety and depressive symptoms, as measured by HADS, with both scores falling below the threshold of clinical significance at 4 months (Yosipovitch et al., 2024). The case series by Lönndahl et al. similarly highlighted that dupilumab treatment was associated with significant improvements in depressive symptoms, pruritus intensity and, subsequently, quality of life (Lönndahl et al., 2022).

Sleep deprivation is among the most debilitating consequences of severe PN as in our patient who reported a prompt restoration of sleep at 6 weeks, after months of near-complete sleep deprivation. This aligns with the pooled PRIME/PRIME2 data, which demonstrated improvements in sleep quality as early as week 4 and maintained through week 24 (Lönndahl et al., 2022).

Our 75-year-old patient tolerated therapy with dupilumab, with no reported adverse reactions. This observation is consistent with the favorable safety profile across different age groups described in the literature, including the elderly with multiple comorbidities (Giura et al., 2020; Paganini et al., 2024) as PN is frequently associated with severe cardiovascular, neurological, gastrointestinal, and renal conditions (Bao et al., 2025). Our case highlights a distinct clinical phenotype, characterized by severe PN disease in the absence of systemic comorbidities.

A particularly noteworthy aspect of our case is the rapidity and multidimensional nature of clinical improvement. After starting dupilumab, our patient experienced a marked reduction in pruritus (NRS 9 to 3), as early as 6 weeks, in association with early restoration of sleep and parallel improvements in quality of life and psychological distress, while other prospective and retrospective clinical studies have demonstrated significant efficacy of dupilumab at a later timepoint (from 24 weeks onwards) (Zhao Y. et al., 2025; Lönndahl et al., 2022; Giura et al., 2020; Paganini et al., 2024). Available clinical evidence on dupilumab efficacy in prurigo nodularis is summarized in Table 1.

**TABLE 1:** Summary of clinical evidence on dupilumab efficacy in prurigo nodularis.

Study type	N	Age	Follow-Up	Pruritus improvement (% ≥4 NRS Reduction)	Skin outcomes (% IGA 0/1)	References
Observational multicenter	73	18–80 years	≥16 weeks	84.9%	46.6%	Zhao Y. et al. (2025)
Retrospective cohort	76	19–82 years	52 weeks	NR	NR	Zhao Z. et al. (2025); Lönndahl et al. (2022)
Real-world retrospective	16	69 ± 18.9 years	Up to 84 weeks	100%	100%	Paganini et al. (2024)
Case-report (elderly)	1	85 years	6 months	NR*	NR*	Giura et al. (2020)

Outcomes were standardized as ≥4-point NRS reduction for pruritus and IGA PN-S 0/1 for skin response when available; otherwise, data were reported as not reported (NR). Evidence includes randomized trials and real-world studies, including long-term follow-up up to 84 weeks.

The case of PN we describe further identifies this disease as a neuroimmune disorder in which type 2-driven inflammation drives both cutaneous inflammation but also neural sensitization and central perception of itch both in atopic and non-atopic cases (Bao et al., 2025).

## 5 Patient perspective

The patient reported a marked and rapid improvement in pruritus following initiation of dupilumab, with a consequent restoration of sleep quality and daily functioning. Prior to treatment, persistent and severe itch significantly impaired quality of life, leading to sleep disturbance and psychological distress. After therapy initiation, the reduction in itch intensity was perceived as substantial, allowing the patient to resume normal daily activities. The improvement in skin lesions was also positively perceived, contributing to enhanced self-esteem and overall wellbeing. The treatment was well tolerated, and the patient expressed high satisfaction with the therapeutic outcome.

## Data Availability

The raw data supporting the conclusions of this article will be made available by the authors, without undue reservation.
